# Melatonin Prescription in Children and Adolescents in Relation to Body Weight and Age

**DOI:** 10.3390/ph16030396

**Published:** 2023-03-06

**Authors:** Elin E. Kimland, Elin Dahlén, Jari Martikainen, Jimmy Célind, Jenny M. Kindblom

**Affiliations:** 1 Swedish Medical Products Agency, 751 03 Uppsala, Sweden; elin.kimland@lakemedelsverket.se (E.E.K.); elin.dahlen@lakemedelsverket.se (E.D.); 2 Department of Clinical Science and Education, Södersjukhuset, Karolinska Institutet, 118 83 Stockholm, Sweden; 3 Bioinformatics and Data Centre, The Sahlgrenska Academy, University of Gothenburg, 405 30 Gothenburg, Sweden; jari.martikainen@gu.se; 4 Institute of Medicine, The Sahlgrenska Academy, University of Gothenburg, 405 30 Gothenburg, Sweden; jimmy.celind@gu.se; 5 Department of Pediatrics, Institute of Clinical Sciences, The Sahlgrenska Academy, University of Gothenburg, 416 85 Gothenburg, Sweden; 6 Department of Drug Treatment, Sahlgrenska University Hospital, Region Västra Götaland, 413 45 Gothenburg, Sweden

**Keywords:** melatonin dose, children, adolescents, overweight, obesity, age

## Abstract

The prescription of melatonin to children and adolescents has increased dramatically in Sweden and internationally during the last ten years. In the present study we aimed to evaluate the prescribed melatonin dose in relation to body weight and age in children. The population-based BMI Epidemiology Study Gothenburg cohort has weight available from school health care records, and information on melatonin prescription through linkage with high-quality national registers. We included prescriptions of melatonin to individuals below 18 years of age where a weight measurement not earlier than three months before, or later than six months after the dispensing date, was available (n = 1554). Similar maximum doses were prescribed to individuals with overweight orobesity as to individuals with normal weight, and to individuals below and above 9 years of age. Age and weight only explained a marginal part of the variance in maximum dose, but were inversely associated and explained a substantial part of the variance in maximum dose per kg. As a result, individuals overweight or with obesity, or age above 9 years, received lower maximum dose per kg of body weight, compared with individuals with normal weight or below 9 years of age. Thus, the prescribed melatonin dose to individuals under 18 years of age is not primarily informed by body weight or age, resulting in substantial differences in prescribed dose per kg of body weight across BMI and age distribution.

## 1. Introduction

Pediatric sleep disorders are common. About one in four, and among individuals with neuropsychiatric disorders an even higher proportion, experience sleeping difficulties at some point during childhood [1,2,3,4,5]. As a result, the prescription of melatonin to children and adolescents has increased dramatically in Sweden [6] and other Nordic countries [7,8] during the last ten years. Melatonin is used for sleep disorders in children and adolescents, and it is recommended in national guidelines as a second line of treatment after non-pharmacological alternatives [9,10]. Although melatonin is recommended as a short-term treatment, several register-based studies show that long-term use is common [6,8,11,12]. These findings indicate a substantial exposure for some individuals where treatment is initiated during childhood and continues long-term. The adverse events following short-term treatment have been reported to be minor, transient, and easily managed, mostly related to fatigue, mood, or psychomotor and neurocognitive performance and therefore melatonin is considered a safe medication [13]. There is no reported withdrawal syndrome, and the risk of acute toxicity appears to be low, even at high doses. It has been discussed that long-term medication with melatonin may influence sexual maturation, although a recent cohort study refuted this [14]. Further investigations are needed before long-term safety of melatonin use starting in childhood or adolescence can be established [13].

Melatonin is metabolized mainly via cytochrome p450 (CYP) 1A2 together with several other psychiatric drugs, and this metabolizing enzyme has fluvoxamine and paroxetine as known inhibitors [15]. These drugs however, are rarely used in children in Sweden.

The authorization status for melatonin varies among countries. In the USA, melatonin is regarded as a dietary supplement, while it is available as an over-the-counter drug or on prescription for adults in several European countries. Since 2018 melatonin is approved for sleep disorders in children with neuropsychiatric comorbidity by the EMA. In most European countries melatonin is available as a prescribed medicine as well as an over-the-counter drug and in some countries also regarded as a dietary supplement. Only authorized medical products are under pharmacovigilance surveillance and good manufacturing practice to guarantee correct substance in an adequate amount and no contamination. Authorized melatonin is available both as an oral solution (1 mg/mL) as well as direct acting tablets in strengths from 1–5 mg and prolonged-release tablets 1–5 mg. Melatonin dosing largely depends on purposes [16]. In children and adolescents, melatonin is labelled for insomnia and as far as sleep and circadian disorders are considered, low doses are considered preferable [16].

Medicines to children are commonly dosed according to weight and/or age to adjust for physiologic and pharmacokinetic differences compared to adults [17]. Growth and development have profound effects on drug disposition in children as a consequence of maturational alterations in drug absorption, distribution, metabolism and elimination, and due to increased weight and changes in body composition [18]. Obesity is associated with additional changes in body composition and physiology that may affect pharmacokinetics of drugs. Increased liver size, hepatic perfusion and glomerular filtration rate have been reported in children with obesity. The rate-limiting pharmacokinetic and physiological variables do not display alterations that are proportionate with the increase in body size [19]. Therefore, the conventional mg/kg dosing may not be applicable in children with obesity. Pediatric pharmacokinetic studies rarely include data for the population of children with obesity and hence, this is an area where knowledge gaps are especially large [19]. For melatonin, the starting dose according to the information in the summary of product characteristics for melatonin products approved by the European Medicines Agency (EMA), as well as recommendations in Nordic national guidelines [9,10], is 1–5 mg in children 4 years of age and older. The further recommendation is to increase the dose until sufficient effect is reached. In line with these national recommendations, the melatonin doses were in the range from 1 to 10 mg in the studies included in a recently published systematic review [20]. However, the optimal dose per kg of body weight, and whether children and adolescents overweight and with obesity, or older children, would benefit from higher doses, has not been established. In the present study we aimed to evaluate the prescription of melatonin in relation to body weight and age.

## 2. Results

In the present study we used the population-based cohort BMI Epidemiology Study Gothenburg to evaluate the prescription of melatonin in relation to body weight and age. In total, 1554 children below 18 years of age were dispensed melatonin at least once during the study period and had a weight measurement available in the required interval. The mean age at prescription was 11.7 years (SD 3.5 years) and 62.7% were males. Overweight or obesity was seen in 24.8% of the individuals in the cohort (Table 1). We found that 77% received a maximum melatonin dose of 5 mg or less, and the most prescribed tablet strengths were 2 mg and 3 mg (Table 1). 1007 (64.8%) individuals received a first prescription while 547 (35.2%) received an iterated prescription.

The maximum dose displayed a range of 1.0–25.0 mg, and the maximum dose in relation to body weight was 0.01–1.53 mg/kg. We observed that the maximum dose, and maximum dose per kg of body weight, were significantly higher among the iterated prescriptions (4.6 mg (SD 3.2) and 0.13 mg/kg body weight (SD 0.15), respectively), than the first prescriptions (3.5 mg (SD 2.2) and 0.09 mg/kg body weight (SD 0.07), respectively, *p* < 0.001 for both). We therefore present the subsequent results divided into first and iterated prescriptions of melatonin.

### 2.1. Maximum Dose of Melatonin

The range of the maximum dose of melatonin was 1–20 mg for first prescriptions and 1–25 mg for iterated prescriptions. Individuals with overweight or obesity were prescribed similar maximum total doses as individuals with normal weight, and children under 9 years of age received similar maximum doses as children above 9 years of age (Table 2). Weight and age (separately) only marginally explained the variance in maximum dose for first or iterated prescriptions (Table S1).

These findings demonstrate that similar maximum doses of melatonin were prescribed irrespective of age and weight.

### 2.2. Maximum Dose per Kg of Body Weight

The range of the maximum prescribed melatonin dose per kg of body weight was 0.01–0.85 mg/kg for first prescriptions and 0.02–1.53 mg/kg for iterated prescriptions. Individuals with overweight or obesity were prescribed significantly lower maximum dose per kg of body weight compared with individuals without overweight or obesity, for both first and iterated prescriptions (Table 2). Children above 9 years of age received significantly lower doses per kg of body weight in both first prescriptions and iterated prescriptions compared to children below 9 years of age (Table 2). Weight was a strong inverse predictor of the maximum dose per kg and explained a substantial part of the variance in dose per kg (Table S1). Similarly, age at prescription showed a strong inverse association with the maximum dose per kg and explained a substantial part of the variance.

Thus, lower doses per kg of body weight were prescribed to individuals with overweight or obesity compared with individuals without elevated BMI, and to individuals over 9 years of age compared to below 9 years of age.

### 2.3. Prescriptions According to Sex, Psychiatric and Neuropsychiatric Co-Morbidities

There were no statistically significant differences in maximum total dose or maximum dose per kg of body weight for girls versus boys, or individuals with or without psychiatric comorbidity in the first or iterated prescriptions (Table 3). There were no significant differences in age or weight between children with and without psychiatric comorbidity. Individuals with neuropsychiatric disorder received lower maximum doses and lower dose per kg of body weight on iterated prescriptions (Table 3).

### 2.4. Prediction of Dose and Dose per Kg

A linear regression model showed that age, weight, sex, psychiatric comorbidity, and neuropsychiatric disorder together explained only a marginal part of the variance in dose (Table S1). For dose per kg however, age and weight explained a substantial part of the variance but the addition of sex, psychiatric comorbidity or neuropsychiatric disorder only marginally increased the variance explained (Table S1).

### 2.5. Dosing in Individuals According to Ideal Body Weight

For children with excess weight, it is sometimes recommended to use the ideal body weight (IBW) to calculate a drug dose. Using the Moore method [22], we calculated the IBW of a girl and boy with obesity based on the mean age and height for the group of girls and boys with obesity in the present study. According to the Swedish reference growth charts [23], the IBW of a 12-year-old girl of 1.51 m was 41 kg, and for a boy of 11.5 years and 1.55 m it was 44 kg. The results demonstrate that the dose per kg of IBW for the average girl and boy with obesity equaled the dose per kg in children without obesity (Table S2).

There was no statistical difference in dose or dose per kg between girls and boys with obesity (Table S2).

## 3. Discussion

In the present study we used the population-based BEST Gothenburg cohort with information on body weight together with data on melatonin prescriptions to study the dispensed maximum dose of melatonin in relation to body weight and age. We found that most of the individuals were prescribed a maximum dose of 5 mg or less but that the dose per kg of body weight displayed a large variability. The main finding was that there was no association between body weight and the maximum dose. Similar maximum doses were prescribed to individuals overweight or with obesity as to individuals without elevated BMI, and to older and younger individuals. As a result, individuals with an elevated BMI or older age received lower maximum dose per kg of body weight. Thus, melatonin prescriptions to individuals under 18 years of age are not primarily informed by body weight or age, resulting in substantial differences in prescribed dose per kg of body weight across BMI and age distribution.

The effectivity of melatonin as treatment of sleep disorders is rather well studied in children with neuropsychiatric disorders. Melatonin in the dose range of 2–10 mg demonstrated improved sleep duration and sleep latency onset in pediatric patients 2–18 years of age with neuropsychiatric disorder in a recent systematic review [20]. In another systematic review, melatonin was shown to be the most effective treatment for sleep disorders together with parental education and behavioral interventions in children with autism spectrum disorder [24]. In children without neuropsychiatric disorders there is less evidence to support the use. One small, randomized study demonstrated statistically significant improvements in sleep parameters with melatonin 5 mg compared with placebo in children 6–12 years of age [25]. Short term use of melatonin has demonstrated good safety [13], but several register-based studies show that long-term use is common [6,12]. Considering the multiple physiological effects of melatonin and the scarce knowledge of long-term safety in the pediatric population [13,26,27], along with an exponential rise in use, it is of great importance for health care professionals, care givers and regulatory authorities to follow up the use of melatonin in the pediatric population. In the present study evaluating melatonin prescription according to body weight and age, we demonstrate that similar doses are prescribed to individuals with and without overweight and obesity, as well as to individuals above and below 9 years of age. As a result, individuals receive highly varying doses per kg of body weight. Moreover, similar maximum dose and dose per kg of body weight were seen for girls and boys, and for individuals with and without psychiatric comorbidity, but individuals with neuropsychiatric disorders were prescribed lower doses compared to individuals without neuropsychiatric disorders on iterated prescriptions.

Pediatric sleep disorders are common and may have a significant negative impact on a child’s development and quality of life, including well-being, learning, growth, behavior, and the regulation of emotions [1]. Adequate treatment is therefore important. Melatonin is recommended as the drug of choice in international and national guidelines when pharmacological treatment is needed [9,10,14]. Although the first line of treatment for pediatric sleep disorders is non-pharmacological, there has been a substantial increase in prescriptions of melatonin for sleep disorders among children and adolescents in the Nordic countries [6,7,8,28,29,30]. The recommendations in the summary of products characteristics for different melatonin products authorized in the EU [15] as well as the national guidelines in Sweden [9], recommend 1–5 mg in children 4 years of age and older as a starting dose, and to further increase the dose up to sufficient effect. The studies included in a recent systematic review used melatonin doses ranging from 1 to 10 mg in children under 18 years of age [20]. This is in line with the results in the present study, where 77% were prescribed a dose of 5 mg or lower. However, the optimal dose per kg of body weight, and whether children and adolescents who are overweight and have obesity or are older in age would benefit from higher doses, has not been established. In the present study, we found that similar doses were used for overweight individuals as for individuals with normal BMI, resulting in higher doses per kg of body weight in normal weight individuals. A recent study assessed the link between BMI and effective daily dose of melatonin in adults. Of note, they found that individuals with a higher BMI needed higher doses of melatonin [31]. The authors present two possible mechanisms that might at least partially explain the need for larger doses: that melatonin distributes into fat mass and thus has a larger distribution in individuals with expanded fat mass, and that a receptor variant associated with weaker melatonin signaling is found more frequently in individuals with obesity [32]. Given the lipophilic nature of melatonin, an altered distribution in individuals with obesity is plausible. The findings in that study, together with the findings in the present study that individuals with elevated BMI receive lower melatonin doses, indicate that there is a need to further evaluate the impact of high BMI on the effective dose of melatonin in children. For older adults, a systematic review evaluated melatonin dosing and concluded that the lowest effective dose should be used [16,33]. However, the dosing in that study displayed a large variation [33], indicating that several individual factors may be of importance for dosing of melatonin, such as pronounced inter-individual differences in melatonin production [34], endogenous melatonin production sensitivity to ambient light [35,36], timing of intake of melatonin and pharmaceutical formulation, which has been described in adults. Moreover, some mechanistic studies performed in cell cultures have indicated that melatonin receptors might become desensitized if exposed to exogenous melatonin over a long period of time [37,38]. Although likely to be of importance in children, there are no studies directly assessing the importance of these factors in the pediatric population.

The strengths with the present study are the population-based nature of our cohort, data on melatonin prescriptions together with weight during childhood and adolescence, and that the coverage of the National Prescribed Drug Register is over 99%. The limitations are that no information is available regarding the effect of the melatonin treatment, possible adverse drug reactions, or whether the individuals who were dispensed melatonin used the medication. Another limitation is the restricted geographical representation (mainly the Gothenburg area).

There is a general lack of knowledge regarding medicines in children, but for the sub-population of children with obesity, this knowledge gap is even larger. With the obesity epidemic there is a huge need to learn more regarding drug disposition in children and youth with obesity and therefore, high-quality clinical studies addressing pharmacodynamics, pharmacokinetics, and optimal dosing in this population are warranted.

## 4. Methods

The population-based BMI Epidemiology Study (BEST) Gothenburg cohort was initiated with the overall aim to study the impact of height, weight, and BMI during childhood and puberty on adult diseases, as previously described [39]. The BEST Gothenburg cohort has developmental height and weight measurements available from school health care and child health care [40], and information on causes of death, diagnoses, and prescribed drugs from high-quality national registers in Sweden. Individuals in the BEST Gothenburg cohort were eligible for the present study if they had at least one prescription of melatonin dispensed before 18 years of age in the National Prescribed Drug Register, and in addition had a weight measurement available in the interval up to 3 months before to 6 months after the melatonin dispensing. Melatonin was defined using the Anatomical Therapeutic Chemical (ATC) code N05CH01 [41].

### 4.1. Exposures

The exposures in the present study were body weight, overweight and obesity status, and age at dispensing. We calculated BMI as weight in kilograms divided by height in meters squared and used the International Taskforce for Obesity’s cutoffs for overweight and obesity for age and sex [21]. Furthermore, using information from the National Patient Register in Sweden, we defined psychiatric and neuropsychiatric disorders according to diagnoses in the International Classification of Diseases ICD) system (F00–F99; F84 and F90–F98, respectively). Psychiatric comorbidity was defined as either presence of an F00–F99 diagnosis before the dispensing date of melatonin, or an anti-depressant, treatment for attention-deficit hyperactivity disorder (ADHD), neuroleptic, anxiolytic, or sedative (other than melatonin) in the National Prescribed Drugs Register, dispensed within 6 months before, or 6 months after, the dispensing date of melatonin. The drug treatments were defined using the following ATC codes: N06A (anti-depressants), N06BA and C02AC02 (treatment for ADHD), N05A (neuroleptics), N05B, R06AD (anxiolytics), and N05C, excluding N05CH01 (sedatives other than melatonin).

### 4.2. Outcomes

The National Prescribed Drugs Register is held by the National Board of Health and Welfare in Sweden and was initiated in 2005. The outcome in the present study was maximum dose of melatonin (mg), and maximum dose of melatonin per kilogram body weight (mg/kg) for the first eligible prescription dispensed before 31 January in 2021. The included first eligible prescription could be either a first or an iterated prescription. We defined a prescription as first if there were no previous melatonin prescriptions for that individual during the study period and required at least one year free of melatonin dispensings. For individuals with several dispensings that fulfilled the inclusion criteria, we included the first eligible dispensing. For the maximum dose, we used the tablet strength and information given with the prescription available as free text in the National Prescribed Drugs Register. Two of the investigators (JC and JMK) reviewed every included prescription and retrieved the maximum prescribed dose. For example, if a study subject was prescribed a 2 mg tablet of melatonin with the free text instruction “One tablet each night, if needed increase to 2–3 tablets”, the maximum dose for that prescription was concluded to be 6 mg. For the maximum dose per kilogram body weight, we used the maximum dose divided by the subject’s body weight (mg/kg) in the interval up to 3 months before to 6 months after the date of the melatonin dispensing. The included prescriptions could be first or iterated prescriptions.

### 4.3. Statistical Analyses

Descriptive statistics for continuous variables are presented using mean and standard deviation (SD), and dichotomous variables using number and percentage. We used a two-sided Welch’s *t*-test (independent samples) to test the difference between groups. The associations between exposures and outcomes were analyzed using linear regression models.

## 5. Conclusions

We demonstrate that melatonin prescriptions to individuals under 18 years of age are not primarily informed by body weight or age, resulting in substantial differences in prescribed dose per kg of body weight across BMI and age distribution.

## Figures and Tables

**Table 1:** Cohort and prescription descriptives.

Cohort Descriptives	Mean (SD)	Range
Age at prescription (years)	11.7 (3.5)	1.1–17.9
Height (cm) (n = 1552)	149.9 (19.7)	81.0–200.5
Weight (kg)	45.6 (19.0)	10.0–128.4
BMI (kg/m^2^) (n = 1552)	19.4 (4.5)	12.0–41.8
	**N (%)**	
Males	975 (62.7)	
Age below 9 years	416 (26.8)	
First prescription	1007 (64.8)	
Overweight and obesity (n = 1551)	385 (24.8)	
Obesity (n = 1551)	119 (7.7)	
Any psychiatric diagnosis	1272 (81.9)	
Neuropsychiatric diagnosis	1011 (65.1)	
Pharmacological treatment for depression	306 (19.7)	
Pharmacological treatment for ADHD	777 (50.0)	
Pharmacological treatment with neuroleptics	151 (9.7)	
Pharmacological treatment with anxiolytics	397 (25.5)	
Pharmacological treatment with sedatives ^#^	89 (5.7)	
Any ^##^ drug treatment (other than melatonin)	1157 (74.5)	
Comorbidity (psychiatric diagnosis or drug treatment ^##^)	1408 (90.6)	
**Prescription Descriptives**	**Mean (SD)**	**Range**
Prescribed maximum dose (mg)	3.9 (2.6)	1.0–25.0
Prescribed dose per kg (mg/kg)	0.10 (0.11)	0.01–1.53
	**N (%)**	
Prescribed tablet any strength	1480 (95.2)	
Prescribed maximum dose 5 mg or below	1200 (77.2)	
Prescribed tablet 1 mg	82 (5.3)	
Prescribed tablet 2 mg	882 (56.8)	
Prescribed tablet 3 mg	398 (25.6)	
Prescribed tablet 4 mg	14 (0.9)	
Prescribed tablet 5 mg	104 (6.7)	
Prescribed oral solution 1 mg/mL	74 (4.8)	

Characteristics of the included individuals (n = 1554 unless otherwise stated) and prescriptions in the BMI Epidemiology Study Gothenburg with at least one prescription of melatonin below 18 years of age, and with height and weight available in the interval ≤ 3 months before and ≤6 months after the melatonin prescription. Overweight and obesity were defined according to the cutoffs defined by the International Obesity Taskforce [21]. We collected information on any psychiatric diagnosis (F00–F99) and neuropsychiatric diagnosis (F84 or F90–F98) before the melatonin prescription, and on prescriptions of anti-depressants (N06A), treatment for attention-deficit hyperactivity disorder (ADHD; N06BA and C02AC02), neuroleptics (N05A), anxiolytics (N05B, R06AD), sedatives other than melatonin (N05C, excluding N05CH01). Psychiatric comorbidity was defined as either presence of an F00–F99 diagnosis and/or any of the ATC-codes mentioned above. ^#^ Other than melatonin. ^##^ Of drug treatments mentioned above.

**Table 2:** Maximum prescribed melatonin total dose and dose per kg of body weight in relation to overweight or obesity and age.

	First Prescription			
	Not Overweight or with Obesity (n = 773) Mean (SD)	Overweight or with Obesity (n = 234) Mean (SD)	Difference between Groups Mean (SE) 95% CI	Effect Size Cohen’s d
Maximum total dose (mg)	3.5 (2.1)	3.6 (2.3) NS	0.13 (0.16) −0.20; 0.46	0.061
Maximum total dose per kg of body weight (mg/kg)	0.10 (0.08)	0.07 (0.05) ***	−0.031 (0.004) −0.040; −0.023	−0.44
	**Age 9 or younger** **(n = 252)**	**Above age 9** **(n = 755)**		
Maximum total dose (mg)	3.4 (2.0)	3.6 (2.2)	0.12 (0.15) −0.18; 0.42	0.05
Maximum total dose per kg of body weight (mg/kg)	0.14 (0.09)	0.08 (0.06) ***	−0.06 (0.006) −0.074; −0.050	−0.90
	**Iterated Prescription**			
	**Not Overweight or with Obesity (n = 395)**	**Overweight or with Obesity (n = 151)**	**Difference between Groups Mean (SD)** **95% CI**	**Effect Size** **Cohen’s d**
Maximum total dose (mg)	4.5 (3.2)	4.7 (3.0) NS	0.26 (0.3) −0.32; 0.84	0.08
Maximum total dose per kg of body weight (mg/kg)	0.14 (0.16)	0.10 (0.11) ***	−0.039 (0.01) −0.063; −0.015	−0.27
	**Age 9 or younger** **(n = 164)**	**Above age 9** **(n = 383)**		
Maximum total dose (mg)	5.0 (3.8)	4.4 (2.9)	−0.63 (0.3) −1.28; 0.015	−0.20
Maximum total dose per kg of body weight (mg/kg)	0.22 (0.22)	0.09 (0.07) ***	−0.13 (0.02) −0.16; −0.09	−0.93

Maximum prescribed melatonin total dose and dose per kg of body weight in relation to being overweight or having obesity according to the cutoffs defined by the International Obesity Taskforce [21]. Values are presented as mean (SD) for the first prescriptions and the iterated prescriptions. Statistically significant differences compared with the left column (not overweight or with obesity, and below nine years of age, respectively) are indicated: *** *p* < 0.001. The difference between groups is given as mean (SE) and the effect size is given as Cohen’s d. SD = standard deviation, CI = confidence interval, SE = standard error.

**Table 3:** Maximum prescribed melatonin total dose and dose per kg of body weight in relation to sex, psychiatric, and neuro-psychiatric co-morbidities.

First Prescription Mean (SD)		
	Boys (n = 635)	Girls (n = 372)
Maximum total dose (mg)	3.6 (2.2)	3.5 (2.2) NS
Maximum total dose per kg of body weight (mg/kg)	0.09 (0.07)	0.09 (0.08) NS
	**Without psychiatric comorbidity (n = 107)**	**With psychiatric comorbidity (n = 900)**
Maximum total dose (mg)	3.4 (2.2)	3.6 (2.2) NS
Maximum total dose per kg of body weight (mg/kg)	0.09 (0.08)	0.09 (0.07) NS
	**Without neuro-psychiatric diagnosis (n = 381)**	**With neuro-psychiatric diagnosis (n = 626)**
Maximum total dose (mg)	3.6 (2.3)	3.5 (2.1) NS
Maximum total dose per kg of body weight (mg/kg)	0.08 (0.07)	0.09 (0.07) *
**Iterated Prescription Mean (SD)**		
	**Boys (n = 340)**	**Girls (n = 207)**
Maximum total dose (mg)	4.6 (3.1)	4.5 (3.3) NS
Maximum total dose per kg of body weight (mg/kg)	0.13 (0.15)	0.12 (0.15) NS
	**Without psychiatric comorbidity (n = 39)**	**With psychiatric comorbidity (n = 508)**
Maximum total dose (mg)	4.1 (2.9)	4.6 (3.2)
Maximum total dose per kg of body weight (mg/kg)	0.12 (0.13)	0.13 (0.15) NS
	**Without neuro-psychiatric diagnosis (n = 162)**	**With neuro-psychiatric diagnosis (n = 385)**
Maximum total dose (mg)	5.0 (4.0)	4.4 (2.7) *
Maximum total dose per kg of body weight (mg/kg)	0.16 (0.23)	0.12 (0.09) *

Maximum prescribed melatonin total dose and dose per kg of body weight in relation to sex, psychiatric co-morbidities (defined as an F00–F99 diagnosis before the prescription, or a prescription of anti-depressants (N06A), treatment for attention-deficit hyperactivity disorder (ADHD; N06BA and C02AC02), neuroleptics (N05A), anxiolytics (N05B, R06AD), or sedatives other than melatonin (N05C, excluding N05CH01)), or neuro-psychiatric co-morbidities (defined as an F84 or F90–F98 diagnosis before the melatonin prescription). Values are presented as mean (SD) for the first prescriptions and the iterated prescriptions. Statistically significant differences compared with the left column (boys, without psychiatric co-morbidity, without neuro-psychiatric co-morbidity, respectively) are indicated: * *p* < 0.05. SD = standard deviation.

## Data Availability

Data is contained within the article and Supplementary Material.

## Supplementary Materials

The following supporting information can be downloaded at: https://www.mdpi.com/1424-8247/16/3/396/s1, Table S1: Linear regression models for prediction of dose and dose per kg; Table S2: Dosing of melatonin according to body weight in individuals with obesity.
