# Supplementary material for: Melatonin Prescription in Children and Adolescents in Relation to Body Weight and Age
Source: Pharmaceuticals (Basel). 2023 Mar 6;16(3):396. doi: 10.3390/ph16030396 (PMC10058986; doi:10.3390/ph16030396)
Supplement: Supplementary file 1 [file pharmaceuticals-16-00396-s001.zip › pharmaceuticals-2211306-supplementary.pdf]

Supplementary Material

Melatonin Prescription in Children and Adolescents in Relation to Body Weight and Age

Elin E. Kimland [1,†], Elin Dahlén [1,2,†], Jari Martikainen [3], Jimmy Célind [4,5] and Jenny M. Kindblom [4,6,*]

1 Swedish Medical Products Agency, 751 03 Uppsala, Sweden

2 Department of Clinical Science and Education, Södersjukhuset, Karolinska Institutet, 118 83 Stockholm, Sweden

3 Bioinformatics and Data Centre, The Sahlgrenska Academy, University of Gothenburg, 405 30 Gothenburg, Sweden

4 Institute of Medicine, The Sahlgrenska Academy, University of Gothenburg, 405 30 Gothenburg, Sweden

5 Department of Pediatrics, Institute of Clinical Sciences, The Sahlgrenska Academy, University of Gothenburg, 416 85 Gothenburg, Sweden

6 Department of Drug Treatment, Sahlgrenska University Hospital, Region Västra Götaland, 413 45 Gothenburg, Sweden

* Correspondence: jenny.kindblom@gu.se

† These authors contributed equally to this work.

Supplementary tables

Table S1. Linear regression models for prediction of dose and dose per kg

**Covariates**<br>**First prescription (n=1007)**	**Age**		**Weight**	
Dose	BETA	R^2	BETA	R^2
- Base model	0.03	0.001	-	-
- Adjusted model 1	0.03	0.002	-	-
- Adjusted model 2	0.03	0.002	-	-
- Adjusted model 3	0.02	0.003	-	-
- Base model	-	-	0.05	0.003
- Adjusted model 1	-	-	0.05	0.003
- Adjusted model 2	-	-	0.05	0.003
- Adjusted model 3	-	-	0.05	0.004
- Adjusted model 4	-0.03	-	0.07	0.005
Dose per kg				
- Base model	-0.42	0.18	-	-
- Adjusted model 1	-0.43	0.18	-	-
- Adjusted model 2	-0.43	0.18	-	-
- Adjusted model 3	-0.44	0.18	-	-
- Base model	-	-	-0.45	0.20
- Adjusted model 1	-	-	-0.45	0.20
- Adjusted model 2	-	-	-0.45	0.20
- Adjusted model 3	-	-	-0.45	0.20
- Adjusted model 4	-0.19	-	-0.31	0.22
**Iterated prescription (n=547)**				
Dose				
- Base model	-0.12	0.01	-	-
- Adjusted model 1	-0.12	0.01	-	-
- Adjusted model 2	-0.12	0.03	-	-
- Adjusted model 3	-0.13	0.04	-	-
- Base model	-	-	-0.08	0.006
- Adjusted model 1	-	-	-0.08	0.006
- Adjusted model 2	-	-	-0.07	0.02
- Adjusted model 3	-	-	-0.08	0.03
- Adjusted model 4	-0.16	-	0.05	0.04
Dose per kg				
- Base model	-0.44	0.20	-	-
- Adjusted model 1	-0.45	0.20	-	-
- Adjusted model 2	-0.46	0.23	-	-
- Adjusted model 3	-0.47	0.24	-	-
- Base model	-	-	-0.44	0.19
- Adjusted model 1	-	-	-0.44	0.19
- Adjusted model 2	-	-	-0.44	0.21
- Adjusted model 3	-	-	-0.44	0.23
- Adjusted model 4	-0.29		-0.22	0.26

Linear regression model for dose and dose per kg, for first and iterated prescriptions, respectively. Base model includes either weight or age, and adjusted models include the subsequent addition of 1) sex, 2) sex and neuropsychiatric disorders, 3) sex, neuropsychiatric disorders and psychiatric comorbidity. In model 4, sex, neuropsychiatric disorders and psychiatric comorbidity are added together with both age and weight. The upper panels are the first prescriptions, the lower panels for iterated prescriptions. The data are standardized BETA and variance (R^2).

Table S2. Dosing of melatonin according to body weight in individuals with obesity

	**Individuals without obesity** (=normal weight and overweight)		**Individuals with obesity**	
	**Girls (n=534)** Mean (SD)	**Boys (n=898)** Mean (SD)	**Girls (n=44)** Mean (SD)	**Boys (n=75)** Mean (SD)
Age	12.6 (3.4)	11.1 (3.4)	11.9 (3.5)	11.4 (3.4)
Height (m)	1.51 (0.2)	1.49 (0.2)	1.51 (0.2)	1.55 (0.2)
Weight (kg)	45.3 (15.7)	42.6 (17.6)	67.7 (20.7)	71.9 (26.7)
Ideal body weight (kg)	NA	NA	41	44
Maximum dose (mg)	3.81 (2.7)	3.90 (2.5)	4.07 (2.3)	4.35 (2.8)
Dose per kg (mg/kg)	0.10 (0.11)	0.11 (0.11)	0.07 (0.06)	0.07 (0.05)
Dose per ideal body weight (mg/kg)	NA	NA	0.10	0.11

Descriptive data for the individuals in the cohort categorized according to sex and obesity. The ideal body weight was estimated based on Swedish reference growth charts [22]. We used the Moore method [21]to calculate the ideal body weight for an average girl and boy according to mean in age and height (41 kg for a girl of 12 years and 1.51 m and 44 kg for a boy of 11 years and 1.55 m). The dose and dose per weight did not differ statistically ($p>0.05$) between girls and boys with obesity.
